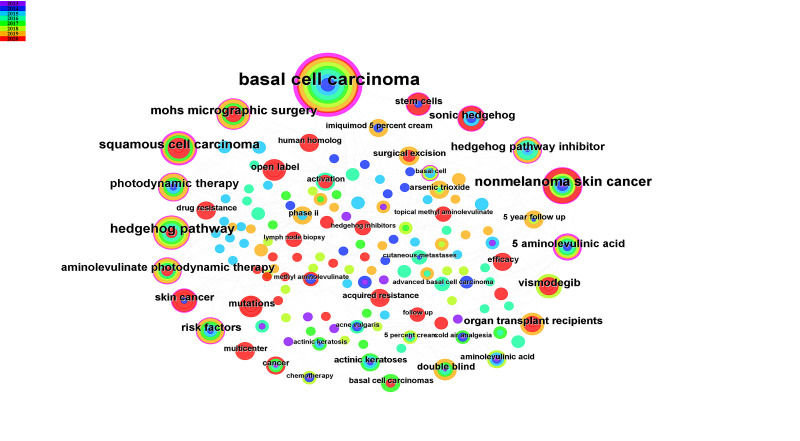# The top 100 most cited articles in the treatment of basal cell carcinoma over the last decade: A bibliometric analysis and review

**DOI:** 10.1097/MD.0000000000037629

**Published:** 2024-04-12

**Authors:** Jinger Lin, Min Luo, Qianwei Zhuo, Haosong Zhang, Nuo Chen, Yue Han

**Affiliations:** a Department of Dermatology, The Union Hospital, Fujian Medical University, Fuzhou, Fujian Province, P.R. China.

**Keywords:** basal cell carcinoma, bibliometrics analysis, hedgehog pathway, treatment

## Abstract

Basal cell carcinoma (BCC) represents the most prevalent cancer globally. The past decade has witnessed significant advancements in BCC treatment, primarily through bibliometric studies. Aiming to perform a comprehensive bibliometric analysis of BCC treatments to comprehend the research landscape and identify trends within this domain, a dataset comprising 100 scientific publications from the Web of Science Core Collection was analyzed. Country co-operation, journal co-citation, theme bursts, keyword co-occurrence, author co-operation, literature co-citation, and field-specific references were examined using VOSviewer and CiteSpace visualization tools. These articles, published between 2013 and 2020, originated predominantly from 30 countries/regions and 159 institutions, with the USA and Germany at the forefront, involving a total of 1118 authors. The keyword analysis revealed significant emphasis on the hedgehog pathway, Mohs micrographic surgery, and photodynamic therapy. The research shows developed nations are at the forefront in advancing BCC therapies, with significant focus on drugs targeting the hedgehog pathway. This treatment avenue has emerged as a crucial area, meriting considerable attention in BCC therapeutic strategies.

## 1. Introduction

Basal cell carcinoma (BCC) originates from epidermal cells and ranks among the most prevalent tumors. BCC incidence is generally higher in men than in women, barring the under-40 female demographic, and typically reaches its zenith around 80 years of age, manifesting as asymptomatic, enlarging, and often hemorrhagic lesions.^[1–3]^ Factors predisposing individuals to BCC encompass UV light exposure, fair skin tone, chronic immunosuppression, male gender, advanced age, and certain genetic skin disorders, culminating in the highest occurrence in Caucasians, with a projected 30% lifetime BCC risk for Caucasian North Americans.^[4]^ Notably, BCC incidence has surged by 5% annually over the previous decade, with Australia reporting the peak incidence rates. In the United States, BCC afflicts 4.3 million individuals yearly, surpassing the combined incidence of all other cancers.^[1]^ Histologically, BCC presents various forms, including noninvasive nodular and superficial subtypes, as well as invasive and infiltrative manifestations typified by morpheiform, infiltrating, micronodular, and basosquamous subtypes.^[5]^ Current guidelines advise the use of topical agents (imiquimod 5%, 5% fluorouracil) and destructive methods (curettage, electrocautery, cryotherapy, laser ablation) for low-risk superficial BCC. Conversely, Mohs micrographic surgery (MMS) is the preferred treatment for high-risk or recurrent BCC and cases involving critical anatomical areas. Hedgehog inhibitors (HPIs) are recommended for patients with locally advanced or metastatic BCC, anti-PD1 antibodies emerge as a promising therapeutic alternative, and Photodynamic therapy (PDT) is recommended for low-risk BCC or as an adjuvant treatment modality.^[6]^

Bibliometrics serves as a method to collect and analyze measurable data from published scientific articles statistically. This approach enables the exploration of trends, research distribution, and pivotal themes within a specific domain.^[7]^ Commonly, in reading or composing academic texts, we reference the work of others. This citation expansion, encompassing forward and backward citation tracing, builds the conceptual framework of knowledge in the field and highlights the importance of the cited research.^[8]^ To our knowledge, no prior study has engaged in a bibliometric analysis of BCC treatments. Hence, this research analyses the top 100 most cited articles from the Web of Science (WOS) database, spanning the last decade. Utilizing 2 bibliometric tools, CiteSpace and VOSviewer, the study aims to enhance the understanding of BCC treatments and concentrate on topical treatments, assisting clinicians in efficiently accessing impactful articles relevant to BCC treatment strategies.

## 2. Materials and methods

### 2.1. Data collection

The Medical Subject Headings (MeSH) database (https://www.ncbi.nlm.nih.gov/mesh/?term=) was queried for MeSH terms “basal cell carcinoma” and “treatment” on August 30, 2023. Subsequent searches using these terms and their free-word equivalents were conducted in the “Core Collection” of WOS. Inclusion criteria were: 1, publication date between 2013 and 2022; 2, article in English; and 3, literature type being review or article. The 100 most cited articles were selected based on citation count, descending. Literature screening was performed by 2 independent evaluators (LJE and LM), with a third party resolving any discrepancies. The final list of top 100 most cited articles, inclusive of author, title, source, sponsor, number of citations, accession number, abstract, address, literature type, designation as a hot paper, and highly cited references, was unanimously agreed upon and downloaded in plain text file format for further analysis.^[9]^ To account for the time lag in accruing citations post-publication, the average citations per year (ACY) metric was calculated using the formula ACY = number of citations/ (2022 – year of publication + 1), facilitating a balanced comparison among scholars.^[10]^

### 2.2. Data analysis

Bibliometric data were processed using VOSviewer (version 1.6.19), CiteSpace (version 6.2.R4 Advanced), Microsoft Office Excel 2019, and mapping tools including SCImago Graphica (version 1.0.36) and Pajek (version 5.18). VOSviewer, a bibliometric mapping software by Nees Jan van Eck and Ludo Waltman, generates a distance-based map, where shorter distances indicate stronger relationships.^[11]^ CiteSpace, visual analytics software designed by Prof Chaomei Chen in 2004, produces graph-based maps and is widely utilized in numerous scientometric analyses.^[12,13]^ Recognizing the unique strengths and limitations of distance-based and graph-based maps, both VOSviewer and CiteSpace were employed to conduct analyses on collaboration networks, citation bursts, inter-country and inter-institutional collaborations, authorship networks, dual-maps of journals, and keyword frequencies and bursts.

## 3. Results

### 3.1. Overview

On August 30, 2023, a thorough search spanning the decade from 2013 to 2022 was conducted in the WOS Core Collection database, yielding 4224 records post-elimination of non-English articles, non-reviews, and non-articles. Title, abstract, and full-text reviews refined these records to the top 100 (T100) most cited articles pertinent to BCC treatments, detailed in Table S1, Supplemental Digital Content, http://links.lww.com/MD/M94 and Figure S1, Supplemental Digital Content, http://links.lww.com/MD/M101.

**Figure 1.**
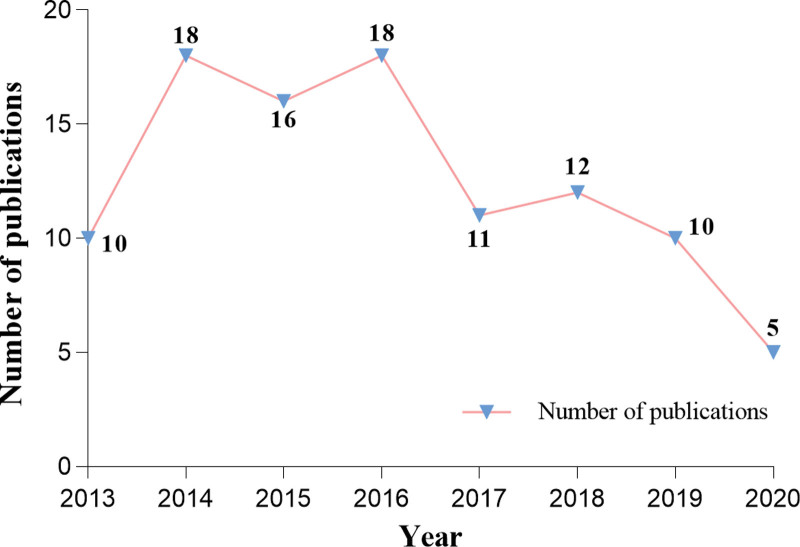
Number of papers published annually between 2013 to 2020. Blue triangular markers represent the publications of every year, pink folds show trend in published articles.

Among the T100, 37 were reviews and 63 were articles; regrettably, no articles from 2021 to 2022 reached the T100. Figure 1 reveals that 2014 and 2016 were peak years, each contributing 18 records.

### 3.2. Reference citation

Citation analysis is a pivotal element of bibliometric studies, serving as an indicator of an article’s impact.^[14]^ WOS is esteemed as a comprehensive research platform across various fields and is globally acknowledged as a reliable citation database by prestigious publishers.^[15]^ Table S1, Supplemental Digital Content, http://links.lww.com/MD/M94 shows the 3 most frequently cited articles revolve around “Photodynamic Therapy,” “Immunotherapy with Anti-PD1 Antibodies,” and “Hedgehog Pathway Therapeutic Potential.” Remarkably, the 2020 article “Clinical Development and Potential of Photothermal and Photodynamic Therapies for Cancer” from Nature Reviews Clinical Oncology not only secured the highest total citation count but also the highest average annual citation rate.

### 3.3. Leading countries/regions

**Figure 2.**
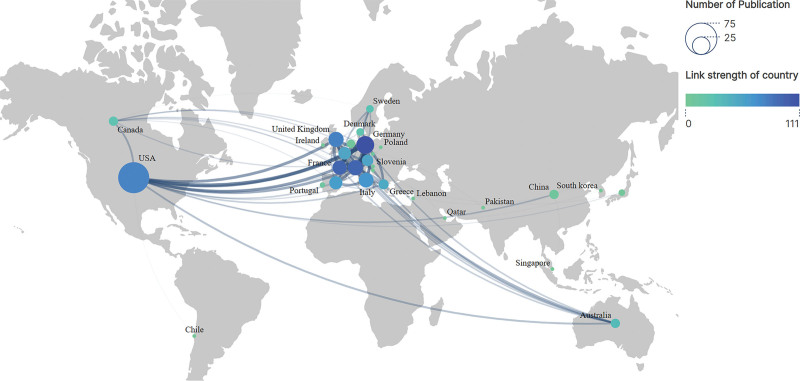
Countries/regions co-operation. Different colored nodes indicate different countries, darker colors indicate more extensive collaboration links with other countries. The line thickness indicates the strength of connections between 2 countries, while the node size reflects the number of articles published in each country. VOSviewer parameters: method (Strength of Link), with a minimum number of country documents set to 1, identified 30 countries/regions. The results include data from 30 countries, all matching the specified threshold.

The United States holds the forefront in the realm of highly cited publications, contributing to 67% of the total, markedly outstripping Germany, the runner-up (Table S2, Supplemental Digital Content, http://links.lww.com/MD/M95). Interestingly, Germany exhibits the most robust international research collaboration, evidenced by a linkage strength of 76, closely trailed by Switzerland and France, both at 94. Among the top 10 countries/regions in Table S2, Supplemental Digital Content, http://links.lww.com/MD/M95, 5 demonstrate a centrality of ≥0.1, underlining their pivotal role in this research area. These include the United States (Centrality: 0.44), Switzerland (Centrality: 0.12), the United Kingdom (Centrality: 0.11), Italy (Centrality: 0.1), and Austria (Centrality: 0.44). In Figure 2, the variation in node color represents different countries, with darker hues indicating denser collaborative networks, Germany being the most prominent. The line thickness denotes the collaboration strength between countries, while node size reflects each country’s publication volume, with the United States having the largest node.

### 3.4. Distribution of institutions

**Figure 3.**
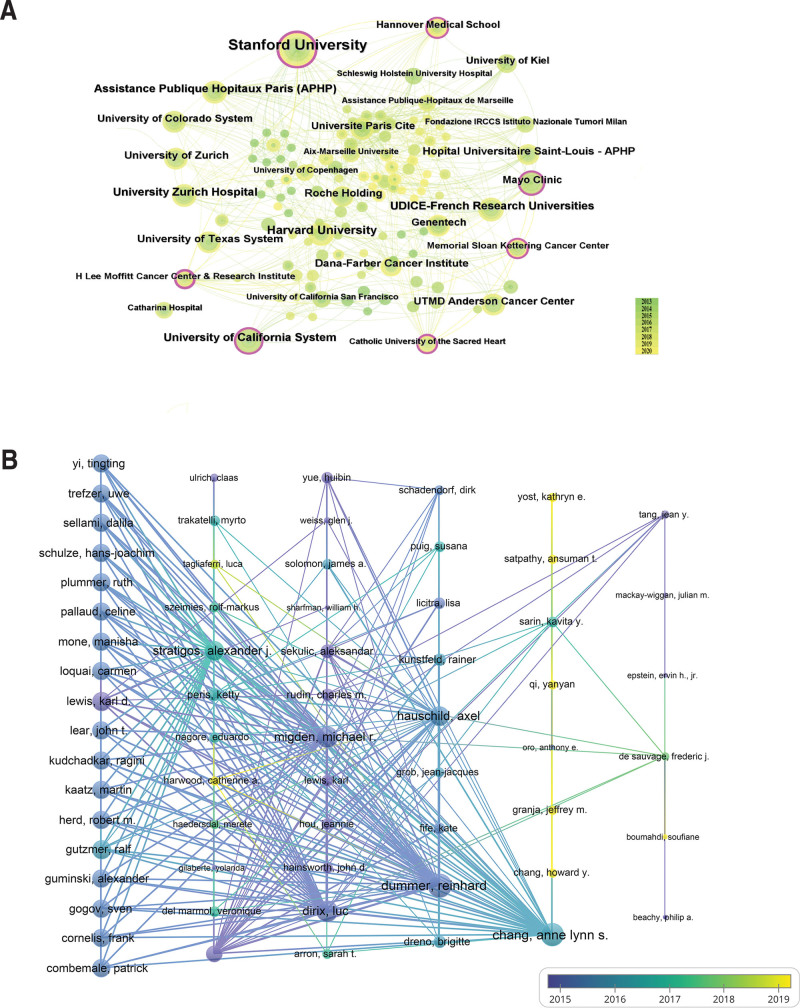
The co-operation of different institutions and authors. (A) Institutions co-operation. Node size reflects co-occurrence frequency, and links indicate collaborations. The colors of the nodes represent different years, with nodes with the outermost purple circle indicating high between-ness centrality (≥0.1). CiteSpace parameters:timespan: 2013 to 2022, slicelength: 1, g-index: k = 25, pruning: pathfinder. (B) Authors co-operation. Node size reflects the co-occurrence frequency and links indicate collaborations. The colors of the nodes represent different years, with blue to yellow indicating 2015 to 2019. VOSviewer parameters: method (Strength of Link), with a minimum number of country documents set to 2. A total of 118 authors were identified.

Table S3, Supplemental Digital Content, http://links.lww.com/MD/M96, enumerates the top ten institutions, with Stanford University leading. This institution published the most papers on BCC treatment in 2015 and 2016, totaling 4, and maintained a consistent output from 2018 to 2020 (Figure S2, Supplemental Digital Content, http://links.lww.com/MD/M102). Notably, Stanford University (Centrality: 0.28) and the University of California System (Centrality: 0.11) are the only institutions with centrality values exceeding 0.1. In Figure 3A, nodes of high centrality (Centrality ≥ 0.1) are encircled by a distinct purple ring, with the ring’s thickness proportional to the centrality value, visually signifying the node’s significance.^[16]^ The color transition from yellow to green spans from 2013 to 2020.

### 3.5. Co-occurrence of authors

The field of BCC treatment has witnessed contributions from a total of 702 authors. As detailed in Table S4, Supplemental Digital Content, http://links.lww.com/MD/M97, Chang, Anne Lynn S., has emerged as the most prolific author with the highest number of publications (n = 9) and the most citations. However, when assessing the strength of collaborative networks, Reinhard Dummer is notably prominent. Although having the second-highest publication count (n = 8), Reinhard Dummer exhibits the most intensive collaborative network among authors. In Figure 3B, a color gradient from blue to yellow depicts the evolution of collaboration over time, pinpointing 2015 to 2017 as a period of particularly robust collaboration among authors.

### 3.6. Journals

Academic journals are instrumental in the dissemination of research findings.^[9]^ The 100 selected articles were published across 243 academic journals. Table S5, Supplemental Digital Content, http://links.lww.com/MD/M98 profiles the top 10 most prolific journals, which collectively published 533 articles, nearly half of the total count. The New England Journal of Medicine is at the forefront with 66 publications, followed closely by The Lancet Oncology (n = 42) and Clinical Oncology (n = 45). Among these top 10 journals, 8 rank in the first quartile (Q1), and 7 boast an Impact Factor (IF) exceeding 10.0, with 7 based in the United States and the rest in the United Kingdom. In terms of co-citations, the leading trio comprises the Journal of the American Academy of Dermatology (9610 citations), British Journal of Dermatology (9510 citations), and Journal of Medicine (9306 citations).

**Figure 4.**
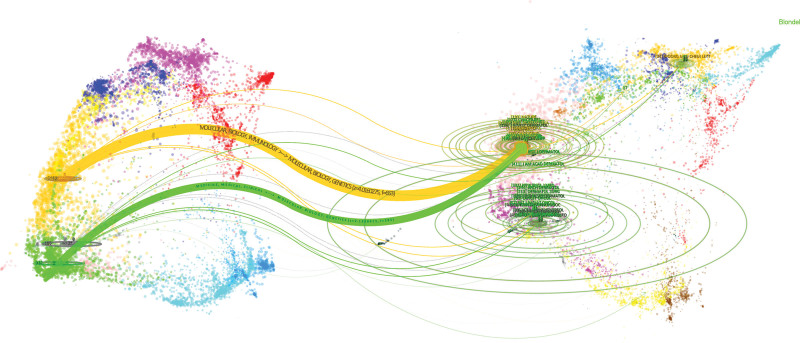
Dual-map overlay analysis of journals. Source journals are represented on the left side and cited journals on the right side. CiteSpace parameters:sources circle size:100, target circle size: 150, snap to centroids: 0.

The dual-map overlay of journals reveals thematic linkages between journals.^[9]^ The map’s left side represents source journals, while the right side portrays the cited journals. Figure 4 delineates 2 primary citation trajectories. The orange pathway indicates that articles from molecular, biological, and genetics journals are predominantly cited by journals in molecular, biological, and immunological sciences; conversely, the green pathway suggests that articles from molecular, biological, and genetics journals are chiefly cited by journals in medicine, medical, and clinical fields. This highlights the centrality of medicine, molecular biology, and genetics within the domain.

### 3.7. Keywords and trend topics

**Figure 5.**
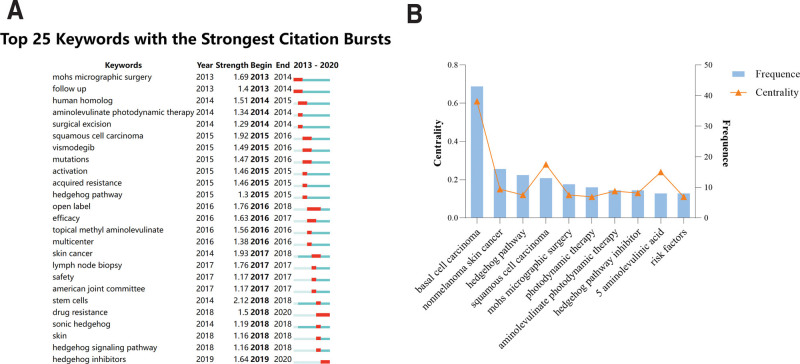
Keywords. (A) Top 25 keywords with the strongest citation bursts. The red area is the range of year in which the keywords were cited more frequently. (B) Top 10 keywords frequently cited and their centrality. Blue bars suggest citation frequency, and orange line graphs show changes in centrality.

The T100 literature encapsulates pivotal keywords, with varying colors denoting different years and larger nodes symbolizing a higher co-occurrence frequency. “BCC” not only boasts the highest occurrence frequency but also the most substantial centrality (0.61) (Figure S3, Supplemental Digital Content, http://links.lww.com/MD/M103). Figure 5 displays the top 25 keywords with the most pronounced citation bursts and the top ten keywords with the highest occurrence frequency over the past decade. A citation burst represents a marked uptick in citation frequency within a specific timeframe.^[17]^ Analysis from Figure 5A reveals that MMS experienced a citation burst during 2013 to 2014, ranking fifth in occurrence frequency (Fig. 5B). Concurrently, “surgical excision” also demonstrated a citation burst in 2014, closely succeeded by PDT, which focused on a citation burst in 2014. Finally, keywords associated with the Hedgehog (Hh) pathway, encompassing Hh signaling pathways and HPIs, were prominent from 2015 to 2020, showcasing high frequency and centrality. This trend analysis indicates a gradual shift in research focus from surgical to nonsurgical treatment modalities.

## 4. Conclusion

BCC typically follows a brief clinical course with a metastatic rate under 1%, yet it can induce significant pain and potentially lead to mortality, particularly in vulnerable groups such as the elderly and immunocompromised individuals.^[17–19]^ Bibliometric analyses of BCC treatments revealed that the most referenced papers and leading institutions hail from developed nations, notably the United States, the United Kingdom, and Germany. The analysis of institutional contributions further illustrated that these countries lead in BCC research, with the top ten institutions located in developed regions like the United States, Switzerland, and France. This prominence is attributed not only to the high prevalence of BCC in Caucasians but also to the superior healthcare resources in these countries, facilitating earlier BCC detection and treatment. The majority of highly cited journals are Q1 publications, suggesting that clinicians should prioritize top-tier journals to stay abreast of the latest BCC research developments.

Standard surgical excision (SSE) remains the benchmark for treating noninvasive BCC. Keyword citation burst analysis indicates that “surgical resection” peaked in popularity in 2014. Impressively, post-SSE, the 5-year disease-free rate reportedly exceeds 98%.^[20]^ A 2021 retrospective study comparing SSE with nonsurgical interventions in 68 BCC patients highlighted SSE’s superior efficacy. Moreover, integrating SSE with repair techniques outperformed nonsurgical and ablative therapies.^[21]^ For high-risk or recurrent BCC, MMS is the optimal surgical choice, boasting cure rates between 94% to 99%.^[3]^ Citation bursts for MMS, ranking fifth in frequency, first emerged in 2013.^[11]^ MMS offers the benefits of minimized incomplete resections and reduced recurrence rates.^[22]^ However, a prospective study on Spanish nonmelanoma skin cancer (NMSC) patients, 92% with BCC, from 2013 to 2020, indicated that 21% of BCC patients treated with MMS faced an elevated risk of a subsequent tumor.^[23]^

Citation analysis, a more robust indicator of article quality and research trends than IF analysis,^[24]^ has highlighted a growing focus on BCC treatments involving the Hh pathway, HPIs, and PDT over the past decade. The Hh pathway is crucial in embryonic development, cell growth regulation, and tissue homeostasis, with around 90% of BCC cases linked to the hyperactivation of Hh proteins.^[20]^ HPIs have risen as a significant treatment for locally advanced BCC (LaBCC) and metastatic BCC (mBCC).^[18,25]^ LaBCC denotes the intricate scenario where a tumor either remains untreated for an extended period or persistently resists and recurs posttreatment, often leading to extensive tissue destruction that makes surgical or radiotherapeutic interventions impracticable.^[1]^ A 2016 retrospective cohort analysis revealed that 0.8% of BCC patients suffer from LaBCC.^[26]^ In mammals, the Hh pathway is constituted by 3 secreted ligands: Sonic Hedgehog (Shh), Indian Hedgehog (Ihh), and Desert Hedgehog (Dhh), the PTCH transmembrane receptor, the SMO signaling transducer, and the GLI transcription factor. PTCH comes in 2 isoforms, PTCH1 and PTCH2, both serving as 12-pass transmembrane Hh receptors. SMO, conversely, functions as a 7-pass transmembrane protein and a G protein-coupled receptor (GPCR). Shh’s interaction with PTCH stimulates SMO, prompting its accumulation and translocation to cilia, which in turn, transcriptionally activates GLI proteins. In Shh’s absence, PTCH1 impedes Hh pathway activation by inhibiting SMO.^[27]^ Notably, 80% to 90% of sporadic BCC tumors harbor PTCH1 mutations, while approximately 10% exhibit SMO mutations.^[28]^

Among HPIs, Vismodegib and Sonidegib have attracted considerable focus. Vismodegib, sanctioned by the US Food and Drug Administration (FDA) in 2012 and the European Medicines Agency, is prescribed for adults with LaBCC or mBCC. It functions by interacting with the 7-transmembrane (7-TM) structural domain of SMO, thereby activating the transcription factor GLI. Vismodegib has demonstrated remission rates of 43% to 60% in LaBCC patients and 30% to 50% in mBCC patients, with early data also indicating an improvement in overall survival.^[2]^ A phase II trial (ClinicalTrials.gov identifier: NCT01201915) revealed that 50% of BCC patients achieved complete clinical clearance, although complete histologic clearance rates were lower than anticipated.^[29]^ Another study (ClinicalTrials.gov identifier: NCT02667574) showcased Vismodegib’s capacity to shrink facial LaBCC tumors, potentially reducing the scope of surgical intervention required.^[30]^ Sonidegib, approved by the FDA in 2015 for LaBCC or mBCC treatment, has proven effective, especially in the elderly demographic (>75 yr), with 86.85% of patients attaining disease control.^[5]^ The BOLT study (ClinicalTrials.gov identifier: NCT01327053), a comprehensive 42-month phase II trial, substantiated the long-term disease control efficacy of Sonidegib in patients with both invasive and noninvasive BCC subtypes. Particularly, invasive BCC and lesion subtypes exhibited the highest objective response rates.^[31]^ PDT has evolved as an exceptionally adaptable treatment for superficial BCC (sBCC) and nodular BCC. 5-aminolevulinic acid-PDT(ALA-PDT) and its variants maintain high clearance rates and low recurrence, but their utility is curtailed by their inability to precisely excise margins, rendering them unsuitable for aggressive and infiltrative BCC types. Moreover, daylight PDT alleviates pain by substituting conventional light sources with daylight, offering a gentler treatment option.^[32]^ In a clinical trial, 98 nBCC lesions, each smaller than 5mm, received treatment with methyl aminolevulinate PDT (MAL-PDT). The trial documented a clearance rate of 89% for the low-risk group and 87% for the high-risk group. Furthermore, the 60-month recurrence-free survival rates were recorded at 82% and 85%, respectively. The combination of PDT with SSE shows promise as an alternative approach to MMS for treatment-resistant BCC. This finding highlights the necessity for continued exploration into the efficacy of PDT in treating BCC.^[33,34]^

Tumor drug resistance poses a formidable obstacle in BCC management. Keyword analysis highlights persistent focus on drug resistance from 2018 to 2020. A case involving a 68-year-old female with a 20-year BCC history revealed multifocal tumor recurrence post 20 weeks of Vismodegib (150 mg/d) treatment, with sequencing uncovering novel heterozygous missense SMO mutations (c.842G > T (p. Trp281Leu) in exon 4 and c.961G > A (p. Val321Met) in exon 5).^[35]^ Similarly, Sonidegib’s binding to SMO was observed to induce SMO mutations, promoting Sonidegib resistance.^[20]^ Itraconazole (ITZ), another HPI, impedes SMO’s localization to cilia, thus inhibiting the Hh pathway. ITZ has demonstrated tumor growth inhibition in tumors resistant to other Hh inhibitors.^[36,37]^ Moreover, the exploration of additional HPIs, including Taladegib, TAK-441, LEQ 506, Imiquimod, and GLI inhibitors, is underway, aiming to establish a potent therapeutic arsenal against BCC.

## 5. Limitation

The scope of this study, focused on analyzing the T100 most cited BCC treatment articles over the past decade, inherently restricts its coverage, particularly lacking recent literature on BCC treatments from 2021 to 2022, potentially omitting emerging trends in the field. Conducted on August 30, 2023, the research might also be subject to data lag, considering the continual updates of databases. Moreover, the study’s reliance on the WOSCC database as the sole information source is a notable constraint. Despite these limitations, the study still offers substantial insights into the general landscape and developmental trends of BCC treatment.

## Acknowledgments

We sincerely thank all the authors who completed the bibliometric analyses with valid records and Professor Chen for designing and providing CiteSpace software.

## Author contributions

**Conceptualization:** Jinger Lin, Min Luo.

**Data curation:** Jinger Lin.

**Methodology:** Jinger Lin.

**Supervision:** Yue Han.

**Visualization:** Jinger Lin, Qianwei Zhuo, Haosong Zhang, Nuo Chen.

**Writing – original draft:** Jinger Lin.

**Writing – review & editing:** Jinger Lin.

## Supplementary Material











**Figure:**
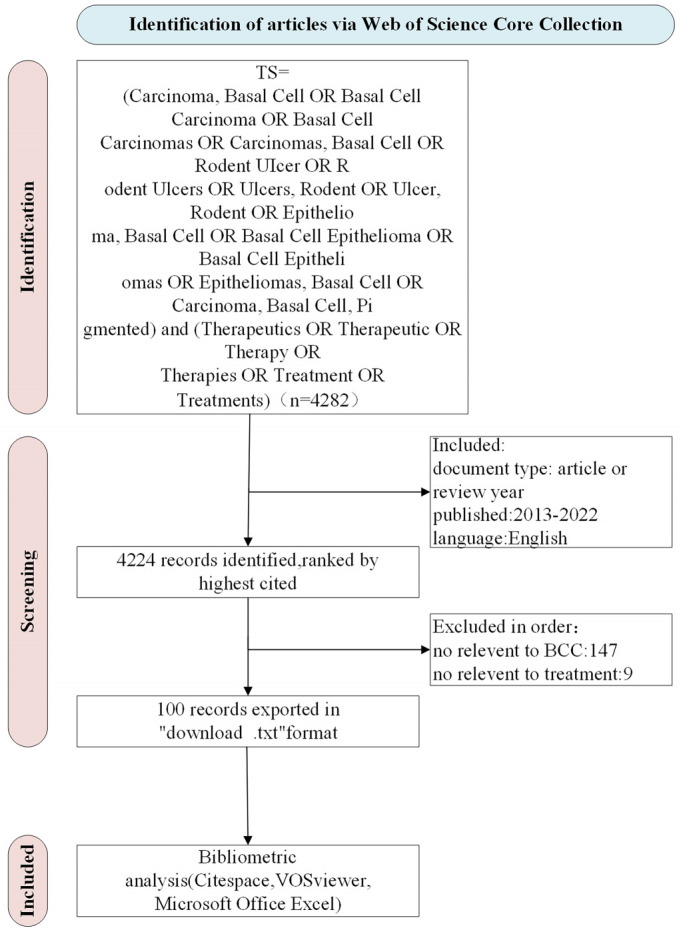


**Figure:**
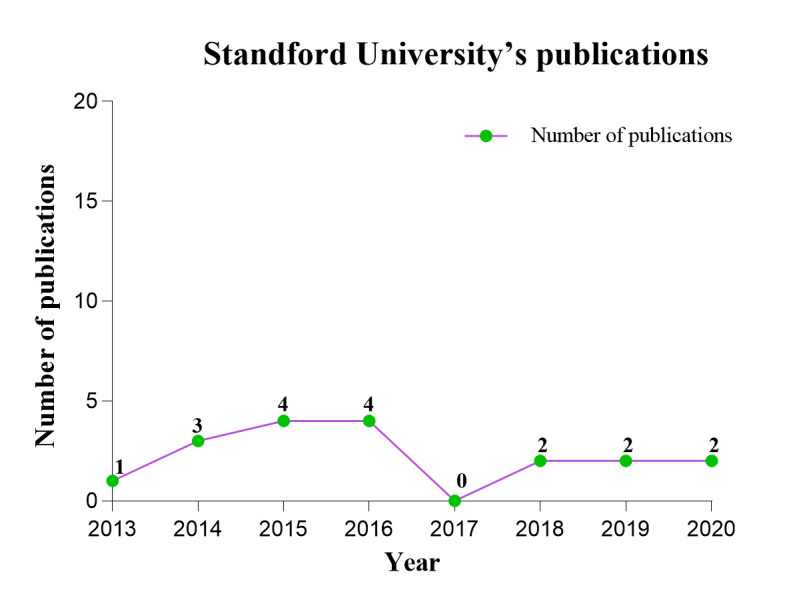


**Figure:**