# Supplementary material for: The top 100 most cited articles in the treatment of basal cell carcinoma over the last decade: A bibliometric analysis and review
Source: Medicine (Baltimore). 2024 Apr 12;103(15):e37629. doi: 10.1097/MD.0000000000037629 (PMC11018215; doi:10.1097/MD.0000000000037629)
Supplement: Supplementary file 1 [file medi-103-e37629-s001.docx]

Table S1 The top 100 cited articles on basal cell carcinoma by ranking.

| Rank | Article | Citations | Year | ACY^a^ |
| --- | --- | --- | --- | --- |
| 1 | [Clinical development and potential of photothermal and photodynamic therapies for cancer](https://www-webofscience-com-443.webvpn.fjmu.edu.cn/wos/woscc/full-record/WOS:000551360100002) | 1210 | 2020 | 403.33 |
| 2 | Clonal replacement of tumor-specific T cells following PD-1 blockade | 668 | 2019 | 167.00 |
| 3 | [Unraveling the therapeutic potential of the Hedgehog pathway in cancer](https://www-webofscience-com-443.webvpn.fjmu.edu.cn/wos/woscc/full-record/WOS:000326920300019) | 419 | 2013 | 41.90 |
| 4 | [Targeting the Sonic Hedgehog Signaling Pathway: Review of Smoothened and GLI Inhibitors](https://www-webofscience-com-443.webvpn.fjmu.edu.cn/wos/woscc/full-record/WOS:000396884700003) | 406 | 2016 | 58.00 |
| 5 | [The role of the Hedgehog signaling pathway in cancer: A comprehensive review](https://www-webofscience-com-443.webvpn.fjmu.edu.cn/wos/woscc/full-record/WOS:000426444900002) | 354 | 2018 | 70.80 |
| 6 | [Massively parallel single-cell chromatin landscapes of human immune cell development and intratumoral T cell exhaustion](https://www-webofscience-com-443.webvpn.fjmu.edu.cn/wos/woscc/full-record/WOS:000482876100024) | 349 | 2019 | 87.25 |
| 7 | [The great escape: tumour cell plasticity in resistance to targeted therapy](https://www-webofscience-com-443.webvpn.fjmu.edu.cn/wos/woscc/full-record/WOS:000511383300015) | 295 | 2020 | 98.33 |
| 8 | [Genomic Analysis of Smoothened Inhibitor Resistance in Basal Cell Carcinoma](https://www-webofscience-com-443.webvpn.fjmu.edu.cn/wos/woscc/full-record/WOS:000350977200006) | 282 | 2015 | 35.25 |
| 9 | [Treatment with two different doses of sonidegib in patients with locally advanced or metastatic basal cell carcinoma (BOLT): a multicentre, randomised, double-blind phase 2 trial](https://www-webofscience-com-443.webvpn.fjmu.edu.cn/wos/woscc/full-record/WOS:000355246600056) | 273 | 2015 | 34.13 |
| 10 | [European guidelines for topical photodynamic therapy part 1: treatment delivery and current indications actinic keratoses, Bowen's disease, basal cell carcinoma](https://www-webofscience-com-443.webvpn.fjmu.edu.cn/wos/woscc/full-record/WOS:000317590500002) | 257 | 2013 | 25.70 |
| 11 | [Diagnosis and treatment of basal cell carcinoma: European consensus-based interdisciplinary guidelines](https://www-webofscience-com-443.webvpn.fjmu.edu.cn/wos/woscc/full-record/WOS:000480414200002) | 237 | 2019 | 59.25 |
| 12 | [Skin Cancer: Epidemiology, Disease Burden, Pathophysiology, Diagnosis, and Therapeutic Approaches](https://www-webofscience-com-443.webvpn.fjmu.edu.cn/wos/woscc/full-record/WOS:000396367500002) | 232 | 2017 | 38.67 |
| 13 | [Epigenetic targeting of Hedgehog pathway transcriptional output through BET bromodomain inhibition](https://www-webofscience-com-443.webvpn.fjmu.edu.cn/wos/woscc/full-record/WOS:000338689500015) | 232 | 2014 | 25.78 |
| 14 | [Hedgehog Signaling: From Basic Biology to Cancer Therapy](https://www-webofscience-com-443.webvpn.fjmu.edu.cn/wos/woscc/full-record/WOS:000397424600005) | 206 | 2017 | 34.33 |
| 15 | [Guidelines of care for the management of basal cell carcinoma](https://www-webofscience-com-443.webvpn.fjmu.edu.cn/wos/woscc/full-record/WOS:000424883000024) | 196 | 2018 | 39.20 |
| 16 | [Open-Label, Exploratory Phase II Trial of Oral Itraconazole for the Treatment of Basal Cell Carcinoma](https://www-webofscience-com-443.webvpn.fjmu.edu.cn/wos/woscc/full-record/WOS:000332483400010) | 194 | 2014 | 21.56 |
| 17 | [GLI activation by atypical protein kinase C iota/lambda regulates the growth of basal cell carcinomas](https://www-webofscience-com-443.webvpn.fjmu.edu.cn/wos/woscc/full-record/WOS:000315661500040) | 192 | 2013 | 19.20 |
| 18 | [Surgical excision versus Mohs' micrographic surgery for basal cell carcinoma of the face: A randomised clinical trial with 10 year follow-up](https://www-webofscience-com-443.webvpn.fjmu.edu.cn/wos/woscc/full-record/WOS:000344628300015) | 190 | 2014 | 21.11 |
| 19 | [Photodynamic therapy versus topical imiquimod versus topical fluorouracil for treatment of superficial basal-cell carcinoma: a single blind, non-inferiority, randomised controlled trial](https://www-webofscience-com-443.webvpn.fjmu.edu.cn/wos/woscc/full-record/WOS:000320371100051) | 187 | 2013 | 18.70 |
| 20 | [Aldara activates TLR7-independent immune defence](https://www-webofscience-com-443.webvpn.fjmu.edu.cn/wos/woscc/full-record/WOS:000318873900014) | 179 | 2013 | 17.90 |
| 21 | [Non-canonical Hedgehog Signaling Pathway in Cancer: Activation of GLI Transcription Factors Beyond Smoothened](https://www-webofscience-com-443.webvpn.fjmu.edu.cn/wos/woscc/full-record/WOS:000471335400001) | 173 | 2019 | 43.25 |
| 22 | [Vismodegib in patients with advanced basal cell carcinoma (STEVIE): a pre-planned interim analysis of an international, open-label trial](https://www-webofscience-com-443.webvpn.fjmu.edu.cn/wos/woscc/full-record/WOS:000355246600057) | 165 | 2015 | 20.63 |
| 23 | [Vismodegib in patients with advanced basal cell carcinoma: Primary analysis of STEVIE, an international, open-label trial](https://www-webofscience-com-443.webvpn.fjmu.edu.cn/wos/woscc/full-record/WOS:000414850400036) | 159 | 2017 | 26.50 |
| 24 | [Update of the European guidelines for basal cell carcinoma management Developed by the Guideline Subcommittee of the European Dermatology Forum](https://www-webofscience-com-443.webvpn.fjmu.edu.cn/wos/woscc/full-record/WOS:000341075100005) | 152 | 2014 | 16.89 |
| 25 | [Photodynamic Therapy: A Clinical Consensus Guide](https://www-webofscience-com-443.webvpn.fjmu.edu.cn/wos/woscc/full-record/WOS:000382555400002) | 150 | 2016 | 21.43 |
| 26 | [Targeting the Hedgehog signaling pathway in cancer: beyond Smoothened](https://www-webofscience-com-443.webvpn.fjmu.edu.cn/wos/woscc/full-record/WOS:000359010000009) | 148 | 2015 | 18.50 |
| 27 | [Pivotal ERIVANCE basal cell carcinoma (BCC) study: 12-month update of efficacy and safety of vismodegib in advanced BCC](https://www-webofscience-com-443.webvpn.fjmu.edu.cn/wos/woscc/full-record/WOS:000354604200031) | 145 | 2015 | 18.13 |
| 28 | [Hedgehog Signaling Restrains Bladder Cancer Progression by Eliciting Stromal Production of Urothelial Differentiation Factors](https://www-webofscience-com-443.webvpn.fjmu.edu.cn/wos/woscc/full-record/WOS:000343343800012) | 141 | 2014 | 15.67 |
| 29 | [Basal Cell Skin Cancer, Version 1.2016](https://www-webofscience-com-443.webvpn.fjmu.edu.cn/wos/woscc/full-record/WOS:000375888500012) | 140 | 2016 | 20.00 |
| 30 | [Photodynamic Therapy](https://www-webofscience-com-443.webvpn.fjmu.edu.cn/wos/woscc/full-record/WOS:000337849300015) | 134 | 2014 | 14.89 |
| 31 | [Surgical excision versus imiquimod 5% cream for nodular and superficial basal-cell carcinoma (SINS): a multicentre, non-inferiority, randomised controlled trial](https://www-webofscience-com-443.webvpn.fjmu.edu.cn/wos/woscc/full-record/WOS:000329135300036) | 134 | 2014 | 14.89 |
| 32 | [Expanded access study of patients with advanced basal cell carcinoma treated with the Hedgehog pathway inhibitor, vismodegib](https://www-webofscience-com-443.webvpn.fjmu.edu.cn/wos/woscc/full-record/WOS:000328694200015) | 133 | 2014 | 14.78 |
| 33 | [Phase I Study of the Hedgehog Pathway Inhibitor IPI-926 in Adult Patients with Solid Tumors](https://www-webofscience-com-443.webvpn.fjmu.edu.cn/wos/woscc/full-record/WOS:000318911600020) | 133 | 2013 | 13.30 |
| 34 | [U.S. Food and Drug Administration Approval: Vismodegib for Recurrent, Locally Advanced, or Metastatic Basal Cell Carcinoma](https://www-webofscience-com-443.webvpn.fjmu.edu.cn/wos/woscc/full-record/WOS:000318361900004) | 129 | 2013 | 12.90 |
| 35 | [Basal cell carcinoma Basal cell carcinoma Contemporary approaches to diagnosis, treatment, and prevention](https://www-webofscience-com-443.webvpn.fjmu.edu.cn/wos/woscc/full-record/WOS:000455473200011) | 127 | 2019 | 31.75 |
| 37 | [Crosstalk between hedgehog and other signaling pathways as a basis for combination therapies in cancer](https://www-webofscience-com-443.webvpn.fjmu.edu.cn/wos/woscc/full-record/WOS:000336825800008) | 124 | 2014 | 13.78 |
| 38 | [Cutaneous adverse effects of targeted therapies Part II: Inhibitors of intracellular molecular signaling pathways](https://www-webofscience-com-443.webvpn.fjmu.edu.cn/wos/woscc/full-record/WOS:000347903200010) | 123 | 2015 | 15.38 |
| 39 | [Metastatic basal cell carcinoma: Prognosis dependent on anatomic site and spread of disease](https://www-webofscience-com-443.webvpn.fjmu.edu.cn/wos/woscc/full-record/WOS:000331715300012) | 122 | 2014 | 13.56 |
| 40 | [Tumor Recurrence 5 Years after Treatment of Cutaneous Basal Cell Carcinoma and Squamous Cell Carcinoma](https://www-webofscience-com-443.webvpn.fjmu.edu.cn/wos/woscc/full-record/WOS:000317698800014) | 120 | 2013 | 12.00 |
| 41 | [Long-term efficacy and safety of sonidegib in patients with locally advanced and metastatic basal cell carcinoma: 30-month analysis of the randomized phase 2 BOLT study](https://www-webofscience-com-443.webvpn.fjmu.edu.cn/wos/woscc/full-record/WOS:000427481900033) | 118 | 2018 | 23.60 |
| 42 | [Smoothened (SMO) receptor mutations dictate resistance to vismodegib in basal cell carcinoma](https://www-webofscience-com-443.webvpn.fjmu.edu.cn/wos/woscc/full-record/WOS:000349582800005) | 113 | 2015 | 14.13 |
| 43 | [Responses of metastatic basal cell and cutaneous squamous cell carcinomas to anti-PD1 monoclonal antibody REGN2810](https://www-webofscience-com-443.webvpn.fjmu.edu.cn/wos/woscc/full-record/WOS:000388042900002) | 112 | 2016 | 16.00 |
| 44 | [European Dermatology Forum Guidelines on topical photodynamic therapy](https://www-webofscience-com-443.webvpn.fjmu.edu.cn/wos/woscc/full-record/WOS:000362902100002) | 112 | 2015 | 14.00 |
| 45 | [European Research on Electrochemotherapy in Head and Neck Cancer (EURECA) project: Results of the treatment of skin cancer](https://www-webofscience-com-443.webvpn.fjmu.edu.cn/wos/woscc/full-record/WOS:000379695900005) | 111 | 2016 | 15.86 |
| 46 | [RAS/MAPK Activation Drives Resistance to Smo Inhibition, Metastasis, and Tumor Evolution in Shh Pathway-Dependent Tumors](https://www-webofscience-com-443.webvpn.fjmu.edu.cn/wos/woscc/full-record/WOS:000361917100018) | 110 | 2015 | 13.75 |
| 47 | [Skin Cancers in Organ Transplant Recipients](https://www-webofscience-com-443.webvpn.fjmu.edu.cn/wos/woscc/full-record/WOS:000411878900006) | 109 | 2017 | 18.17 |
| 48 | [Skin Cancer Epidemics in the Elderly as An Emerging Issue in Geriatric Oncology](https://www-webofscience-com-443.webvpn.fjmu.edu.cn/wos/woscc/full-record/WOS:000411928900011) | 108 | 2017 | 18.00 |
| 49 | [Two intermittent vismodegib dosing regimens in patients with multiple basal-cell carcinomas (MIKIE): a randomised, regimen-controlled, double-blind, phase 2 trial](https://www-webofscience-com-443.webvpn.fjmu.edu.cn/wos/woscc/full-record/WOS:000396344600051) | 107 | 2017 | 17.83 |
| 50 | [Drug Delivery Nanoparticles in Skin Cancers](https://www-webofscience-com-443.webvpn.fjmu.edu.cn/wos/woscc/full-record/WOS:000338879800001) | 107 | 2014 | 11.89 |
| 51 | [Consensus for Nonmelanoma Skin Cancer Treatment: Basal Cell Carcinoma, Including a Cost Analysis of Treatment Methods](https://www-webofscience-com-443.webvpn.fjmu.edu.cn/wos/woscc/full-record/WOS:000354115200002) | 104 | 2015 | 13.00 |
| 52 | [Imiquimod in dermatology: an overview](https://www-webofscience-com-443.webvpn.fjmu.edu.cn/wos/woscc/full-record/WOS:000380353000015) | 103 | 2016 | 14.71 |
| 53 | [Role and Therapeutic Targeting of the PI3K/Akt/mTOR Signaling Pathway in Skin Cancer: A Review of Current Status and Future Trends on Natural and Synthetic Agents Therapy](https://www-webofscience-com-443.webvpn.fjmu.edu.cn/wos/woscc/full-record/WOS:000484537500036) | 100 | 2019 | 25.00 |
| 54 | [Anti-inflammatory and anticancer activities of Naringenin-loaded liquid crystalline nanoparticles in vitro](https://www-webofscience-com-443.webvpn.fjmu.edu.cn/wos/woscc/full-record/WOS:000593653800001) | 99 | 2021 | 49.50 |
| 55 | [Tip-loaded fast-dissolving microneedle patches for photodynamic therapy of subcutaneous tumor](https://www-webofscience-com-443.webvpn.fjmu.edu.cn/wos/woscc/full-record/WOS:000444238000019) | 99 | 2018 | 19.80 |
| 56 | [Metastatic basal cell carcinoma with amplification of PD-L1: exceptional response to anti-PD1 therapy](https://www-webofscience-com-443.webvpn.fjmu.edu.cn/wos/woscc/full-record/WOS:000413239300001) | 99 | 2016 | 14.14 |
| 57 | [The 12-month analysis from Basal Cell Carcinoma Outcomes with LDE225 Treatment (BOLT): A phase II, randomized, double-blind study of sonidegib in patients with advanced basal cell carcinoma](https://www-webofscience-com-443.webvpn.fjmu.edu.cn/wos/woscc/full-record/WOS:000378093300030) | 99 | 2016 | 14.14 |
| 58 | [GEC-ESTRO ACROP recommendations in skin brachytherapy](https://www-webofscience-com-443.webvpn.fjmu.edu.cn/wos/woscc/full-record/WOS:000429762700001) | 98 | 2018 | 19.60 |
| 59 | [Azathioprine and Risk of Skin Cancer in Organ Transplant Recipients: Systematic Review and Meta-Analysis](https://www-webofscience-com-443.webvpn.fjmu.edu.cn/wos/woscc/full-record/WOS:000388208600020) | 98 | 2016 | 14.00 |
| 60 | [Basal cell carcinoma: PD-L1/PD-1 checkpoint expression and tumor regression after PD-1 blockade](https://www-webofscience-com-443.webvpn.fjmu.edu.cn/wos/woscc/full-record/WOS:000396916400003) | 97 | 2017 | 16.17 |
| 61 | [Molecular Pathways: Novel Approaches for Improved Therapeutic Targeting of Hedgehog Signaling in Cancer Stem Cells](https://www-webofscience-com-443.webvpn.fjmu.edu.cn/wos/woscc/full-record/WOS:000348908500004) | 97 | 2015 | 12.13 |
| 62 | [Translational medicine in the field of ablative fractional laser (AFXL)-assisted drug delivery: A critical review from basics to current clinical status](https://www-webofscience-com-443.webvpn.fjmu.edu.cn/wos/woscc/full-record/WOS:000374117100030) | 96 | 2016 | 13.71 |
| 63 | [An Investigator-Initiated Open-Label Trial of Sonidegib in Advanced Basal Cell Carcinoma Patients Resistant to Vismodegib](https://www-webofscience-com-443.webvpn.fjmu.edu.cn/wos/woscc/full-record/WOS:000373358900006) | 95 | 2016 | 13.57 |
| 64 | [Locally enhanced chemotherapy by electroporation: clinical experiences and perspective of use of electrochemotherapy](https://www-webofscience-com-443.webvpn.fjmu.edu.cn/wos/woscc/full-record/WOS:000335781900025) | 95 | 2014 | 10.56 |
| 65 | [Aspects of dosimetry and clinical practice of skin brachytherapy: The American Brachytherapy Society working group report](https://www-webofscience-com-443.webvpn.fjmu.edu.cn/wos/woscc/full-record/WOS:000366540000015) | 94 | 2015 | 11.75 |
| 66 | [An investigator-initiated open-label clinical trial of vismodegib as a neoadjuvant to surgery for high-risk basal cell carcinoma](https://www-webofscience-com-443.webvpn.fjmu.edu.cn/wos/woscc/full-record/WOS:000343918200035) | 94 | 2014 | 10.44 |
| 67 | [TERT Promoter Mutations in Skin Cancer: The Effects of Sun Exposure and X-Irradiation](https://www-webofscience-com-443.webvpn.fjmu.edu.cn/wos/woscc/full-record/WOS:000339126100026) | 94 | 2014 | 10.44 |
| 68 | [Photodynamic therapy and pain: A systematic review](https://www-webofscience-com-443.webvpn.fjmu.edu.cn/wos/woscc/full-record/WOS:000412252100049) | 91 | 2017 | 15.17 |
| 69 | [European Dermatology Forum guidelines on topical photodynamic therapy 2019 Part 1: treatment delivery and established indications - actinic keratoses, Bowen's disease and basal cell carcinomas](https://www-webofscience-com-443.webvpn.fjmu.edu.cn/wos/woscc/full-record/WOS:000499058000036) | 89 | 2019 | 22.25 |
| 70 | [Definitive and Postoperative Radiation Therapy for Basal and Squamous Cell Cancers of the Skin: Executive Summary of an American Society for Radiation Oncology Clinical Practice Guideline](https://www-webofscience-com-443.webvpn.fjmu.edu.cn/wos/woscc/full-record/WOS:000507486200012) | 87 | 2020 | 29.00 |
| 71 | Updates on the Management of Non-Melanoma Skin Cancer (NMSC) | 87 | 2017 | 14.50 |
| 72 | [Characterization and Management of Hedgehog Pathway Inhibitor-Related Adverse Events in Patients With Advanced Basal Cell Carcinoma](https://www-webofscience-com-443.webvpn.fjmu.edu.cn/wos/woscc/full-record/WOS:000386483400010) | 86 | 2016 | 12.29 |
| 73 | [Skin Cancer Epidemiology, Detection, and Management](https://www-webofscience-com-443.webvpn.fjmu.edu.cn/wos/woscc/full-record/WOS:000364727500012) | 85 | 2015 | 10.63 |
| 74 | [Plant derived anticancer agents: A green approach towards skin cancers](https://www-webofscience-com-443.webvpn.fjmu.edu.cn/wos/woscc/full-record/WOS:000433328800194) | 84 | 2018 | 16.80 |
| 75 | [Combined Treatments with Photodynamic Therapy for Non-Melanoma Skin Cancer](https://www-webofscience-com-443.webvpn.fjmu.edu.cn/wos/woscc/full-record/WOS:000364232100145) | 84 | 2015 | 10.50 |
| 76 | [Five-Year Results of a Randomized Controlled Trial Comparing Effectiveness of Photodynamic Therapy, Topical Imiquimod, and Topical 5-Fluorouracil in Patients with Superficial Basal Cell Carcinoma](https://www-webofscience-com-443.webvpn.fjmu.edu.cn/wos/woscc/full-record/WOS:000425749900022) | 81 | 2018 | 16.20 |
| 77 | [European guidelines for topical photodynamic therapy part 2: emerging indications - field cancerization, photorejuvenation and inflammatory/infective dermatoses](https://www-webofscience-com-443.webvpn.fjmu.edu.cn/wos/woscc/full-record/WOS:000318638700002) | 79 | 2013 | 7.90 |
| 78 | [A cell identity switch allows residual BCC to survive Hedgehog pathway inhibition](https://www-webofscience-com-443.webvpn.fjmu.edu.cn/wos/woscc/full-record/WOS:000447807100062) | 78 | 2018 | 15.60 |
| 79 | [Surgery Versus 5% Imiquimod for Nodular and Superficial Basal Cell Carcinoma: 5-Year Results of the SINS Randomized Controlled Trial](https://www-webofscience-com-443.webvpn.fjmu.edu.cn/wos/woscc/full-record/WOS:000394933600017) | 78 | 2017 | 13.00 |
| 80 | [Photodynamic Therapy for Non-Melanoma Skin Cancers](https://www-webofscience-com-443.webvpn.fjmu.edu.cn/wos/woscc/full-record/WOS:000396891400004) | 78 | 2016 | 11.14 |
| 81 | [Chemoprevention of Basal and Squamous Cell Carcinoma With a Single Course of Fluorouracil, 5%, Cream A Randomized Clinical Trial](https://www-webofscience-com-443.webvpn.fjmu.edu.cn/wos/woscc/full-record/WOS:000425134900006) | 77 | 2018 | 15.40 |
| 82 | [Basal Cell Carcinoma: An Evidence-Based Treatment Update](https://www-webofscience-com-443.webvpn.fjmu.edu.cn/wos/woscc/full-record/WOS:000344601300005) | 77 | 2014 | 8.56 |
| 83 | [Application of Intense Pulsed Light in the Treatment of Dermatologic Disease: A Systematic Review](https://www-webofscience-com-443.webvpn.fjmu.edu.cn/wos/woscc/full-record/WOS:000333768600001) | 77 | 2014 | 8.56 |
| 84 | [A slow-cycling LGR5 tumour population mediates basal cell carcinoma relapse after therapy](https://www-webofscience-com-443.webvpn.fjmu.edu.cn/wos/woscc/full-record/WOS:000447807100063) | 76 | 2018 | 15.20 |
| 85 | [Strategies to target the Hedgehog signaling pathway for cancer therapy](https://www-webofscience-com-443.webvpn.fjmu.edu.cn/wos/woscc/full-record/WOS:000430126600004) | 76 | 2018 | 15.20 |
| 86 | [Hedgehog Pathway Inhibitors Promote Adaptive Immune Responses in Basal Cell Carcinoma](https://www-webofscience-com-443.webvpn.fjmu.edu.cn/wos/woscc/full-record/WOS:000352071200009) | 76 | 2015 | 9.50 |
| 87 | [Treatment efficacy with electrochemotherapy: A multi-institutional prospective observational study on 376 patients with superficial tumors](https://www-webofscience-com-443.webvpn.fjmu.edu.cn/wos/woscc/full-record/WOS:000390503100020) | 74 | 2016 | 10.57 |
| 88 | [Inhibition of the hedgehog pathway in patients with basal-cell nevus syndrome: final results from the multicentre, randomised, double-blind, placebo-controlled, phase 2 trial](https://www-webofscience-com-443.webvpn.fjmu.edu.cn/wos/woscc/full-record/WOS:000389537700040) | 74 | 2016 | 10.57 |
| 89 | [Three-Year Follow-Up Results of Photodynamic Therapy vs. Imiquimod vs. Fluorouracil for Treatment of Superficial Basal Cell Carcinoma: A Single-Blind, Noninferiority, Randomized Controlled Trial](https://www-webofscience-com-443.webvpn.fjmu.edu.cn/wos/woscc/full-record/WOS:000380585200081) | 74 | 2016 | 10.57 |
| 90 | [Basal Cell Carcinoma Review](https://www-webofscience-com-443.webvpn.fjmu.edu.cn/wos/woscc/full-record/WOS:000454661800004) | 73 | 2019 | 18.25 |
| 91 | [Basal Cell Carcinoma-Treatments for the Commonest Skin Cancer](https://www-webofscience-com-443.webvpn.fjmu.edu.cn/wos/woscc/full-record/WOS:000338450400001) | 73 | 2014 | 8.11 |
| 92 | [Long-term efficacy and safety of sonidegib in patients with advanced basal cell carcinoma: 42-month analysis of the phase II randomized, double-blind BOLT study](https://www-webofscience-com-443.webvpn.fjmu.edu.cn/wos/woscc/full-record/WOS:000501484400001) | 72 | 2020 | 24.00 |
| 93 | [Hedgehog Signaling and Truncated GLI1 in Cancer](https://www-webofscience-com-443.webvpn.fjmu.edu.cn/wos/woscc/full-record/WOS:000580296400001) | 71 | 2020 | 23.67 |
| 94 | [FDA Approval Summary: Sonidegib for Locally Advanced Basal Cell Carcinoma](https://www-webofscience-com-443.webvpn.fjmu.edu.cn/wos/woscc/full-record/WOS:000401254300001) | 71 | 2017 | 11.83 |
| 95 | [Treatment of Nonfatal Conditions at the End of Life Nonmelanoma Skin Cancer](https://www-webofscience-com-443.webvpn.fjmu.edu.cn/wos/woscc/full-record/WOS:000320050200016) | 71 | 2013 | 7.10 |
| 96 | [Keratinocyte Carcinomas: Current Concepts and Future Research Priorities](https://www-webofscience-com-443.webvpn.fjmu.edu.cn/wos/woscc/full-record/WOS:000464654200006) | 70 | 2019 | 17.50 |
| 97 | [Hedgehog Pathway Inhibitor Therapy for Locally Advanced and Metastatic Basal Cell Carcinoma A Systematic Review and Pooled Analysis of Interventional Studies](https://www-webofscience-com-443.webvpn.fjmu.edu.cn/wos/woscc/full-record/WOS:000379590100015) | 70 | 2016 | 10.00 |
| 98 | [Increased Risk of Cutaneous Squamous Cell Carcinoma After Vismodegib Therapy for Basal Cell Carcinoma](https://www-webofscience-com-443.webvpn.fjmu.edu.cn/wos/woscc/full-record/WOS:000375795400007) | 70 | 2016 | 10.00 |
| 99 | [Hedgehog signaling inhibitors in solid and hematological cancers](https://www-webofscience-com-443.webvpn.fjmu.edu.cn/wos/woscc/full-record/WOS:000474499100005) | 69 | 2019 | 17.25 |
| 100 | [Hedgehog Signaling Pathway and Autophagy in Cancer](https://www-webofscience-com-443.webvpn.fjmu.edu.cn/wos/woscc/full-record/WOS:000442869800131) | 69 | 2018 | 13.80 |
|  | [First-in-human trial of nanoelectroablation therapy for basal cell carcinoma: proof of method](https://www-webofscience-com-443.webvpn.fjmu.edu.cn/wos/woscc/full-record/WOS:000330281700016) | 69 | 2014 | 7.67 |

ACY^a^:average citations per year
