# Supplementary material for: The top 100 most cited articles in the treatment of basal cell carcinoma over the last decade: A bibliometric analysis and review
Source: Medicine (Baltimore). 2024 Apr 12;103(15):e37629. doi: 10.1097/MD.0000000000037629 (PMC11018215; doi:10.1097/MD.0000000000037629)
Supplement: Supplementary file 2 [file medi-103-e37629-s002.docx]

Table S2 Ranking of top-10 countries/regions had published the most articles.

| Rank | Country/Region | Article counts | Centrality^b^ | Citations | Total link strength^c^ |
| --- | --- | --- | --- | --- | --- |
| 1 | USA | 67 | 0.44 | 10252 | 76 |
| 2 | Germany | 22 | 0.08 | 3019 | 111 |
| 3 | Switzerland | 16 | 0.12 | 2230 | 94 |
| 4 | United Kingdom | 16 | 0.11 | 2058 | 79 |
| 5 | Italy | 15 | 0.1 | 1879 | 65 |
| 6 | France | 14 | 0.05 | 2073 | 94 |
| 7 | Spain | 12 | 0.09 | 1478 | 59 |
| 8 | Belgium | 11 | 0.02 | 1702 | 53 |
| 9 | Scotland | 10 | 0.01 | 1600 | 58 |
| 10 | Austria | 6 | 0.44 | 1302 | 54 |

Centrality^b^:calculated by CiteSpace,Total link strength^c^:calculated by VOSviewer
