# Supplementary material for: The top 100 most cited articles in the treatment of basal cell carcinoma over the last decade: A bibliometric analysis and review
Source: Medicine (Baltimore). 2024 Apr 12;103(15):e37629. doi: 10.1097/MD.0000000000037629 (PMC11018215; doi:10.1097/MD.0000000000037629)
Supplement: Supplementary file 3 [file medi-103-e37629-s003.docx]

Table S3 Ranking of top-10 institutions had published the most articles.

| Rank | Institutions | Article counts | | Centrality^d^ | Citations |
| --- | --- | --- | --- | --- | --- |
| 1 | Stanford University | 20 | 0.28 | | 3536 |
| 2 | Harvard University | 10 | 0.05 | | 1304 |
| 3 | Assistance Publique Hopitaux Paris (APHP) | 9 | 0.09 | | 1404 |
| 4 | UDICE-French Research Universities | 9 | 0.02 | | 1425 |
| 5 | University Zurich Hospital | 9 | 0.08 | | 1316 |
| 6 | University of California System | 9 | 0.11 | | 1185 |
| 7 | Hopital Universitaire Saint-Louis - APHP | 8 | 0.02 | | 1318 |
| 8 | Dana-Farber Cancer Institute | 8 | 0.06 | | 1138 |
| 9 | Roche Holding | 8 | 0.08 | | 1408 |
| 10 | University of Texas System | 8 | 0 | | 1260 |

Centrality^d^:calculated by CiteSpace.
