# Supplementary material for: The top 100 most cited articles in the treatment of basal cell carcinoma over the last decade: A bibliometric analysis and review
Source: Medicine (Baltimore). 2024 Apr 12;103(15):e37629. doi: 10.1097/MD.0000000000037629 (PMC11018215; doi:10.1097/MD.0000000000037629)
Supplement: Supplementary file 4 [file medi-103-e37629-s004.docx]

| Rank | Authors | Articles counts | | | Citations | Total link strength^e^ |
| --- | --- | --- | --- | --- | --- | --- |
| 1 | Chang,Anne lynn s. | | 9 | | 1942 | 72 |
| 2 | Dummer,Reinhard | | 8 | | 1237 | 77 |
| 3 | Hauschild,Axel | | | 7 | 935 | 50 |
| 4 | Basset-seguin,Nicole | | 6 | | 919 | 37 |
| 5 | Dirix,Luc | | 4 | | 799 | 61 |
| 6 | Gutzmer,Ralf | | 4 | | 514 | 46 |
| 7 | Kelleners-smeets,Nicole w. j. | | 4 | | 532 | 33 |
| 8 | Migden,Michael r. | | 4 | | 799 | 61 |
| 9 | Mosterd,Klara | | 4 | | 532 | 33 |
| 10 | Nelemans,Patty j. | | 4 | | 532 | 33 |

Table S4 Ranking of top-10 authors had published the most articles.

Total link strength^e^:calculated by VOSviewer
