# Supplementary material for: The top 100 most cited articles in the treatment of basal cell carcinoma over the last decade: A bibliometric analysis and review
Source: Medicine (Baltimore). 2024 Apr 12;103(15):e37629. doi: 10.1097/MD.0000000000037629 (PMC11018215; doi:10.1097/MD.0000000000037629)
Supplement: Supplementary file 5 [file medi-103-e37629-s005.docx]

| Rank | Journal | Article counts | Country | Centrality^f^ | Citations | IF^g^(2022) | JCR^h^ (2022) |
| --- | --- | --- | --- | --- | --- | --- | --- |
| 1 | Journal of the American Academy of Dermatology | 73 | USA | 0.02 | 9610 | 13.8 | Q1 |
| 2 | British Journal of Dermatology | 69 | UK | 0 | 9510 | 10.3 | Q1 |
| 3 | New England Journal of Medicine | 66 | UK | 0.01 | 9306 | 158.5 | Q1 |
| 4 | Clinical Cancer Research | 53 | USA | 0.03 | 8266 | 11.5 | Q1 |
| 5 | Archives of Dermatology | 53 | USA | 0.01 | 6885 | 3 | Q2 |
| 6 | Journal of Investigative Dermatology | 48 | USA | 0 | 6293 | 6.5 | Q1 |
| 7 | Journal of Clinical Oncology | 45 | USA | 0.02 | 7293 | 45.3 | Q1 |
| 8 | Cancer Research | 42 | USA | 0.03 | 7037 | 11.2 | Q1 |
| 9 | Dermatologic Surgery | 42 | USA | 0.01 | 5830 | 2.4 | Q3 |
| 10 | The Lancet Oncology | 42 | UK | 0.01 | 6277 | 51.1 | Q1 |

Table S5 Ranking of top-10 journals had published the most articles.

Centrality^f^:calculated by CiteSpace,IF^g^:Impact factor,JCR^h^:Journal Citation Reports
